# Comparison of historical and current temperatures in show caves (Slovenia)

**DOI:** 10.1007/s42452-021-04881-1

**Published:** 2021-12-04

**Authors:** Stanka Šebela, Janez Turk

**Affiliations:** 1 Znanstvenoraziskovalni Center. Slovenske Akademije Znanosti in Umetnosti, Novi trg 2, 1000 Ljubljana, Slovenia; 2 ZRC SAZU IZRK, Titov trg 2, 6230 Postojna, Slovenia; 3 ZAG, Dimičeva ulica 12, 1000 Ljubljana, Slovenia

**Keywords:** Cave air temperature, Meteorological monitoring, Postojnska Jama, Predjama, Škocjanske Jame, Slovenia

## Abstract

Historical air temperatures at three karst caves in Slovenia have been compared to current data time series. In Postojnska Jama (PJ), the most visited show cave in Slovenia, the significant temperature difference between historical and modern measurements at the Pulpito site relates to the months April to November. Mean monthly temperatures measured at the Sepolcro site (PJ) in the modern period (2016–2019) are year round significantly higher than in the historical period (1935–1937). The temperature increase over the last 85 years in PJ is attributed to outside temperature rise and additional heat input from visitors, especially for Sepolcro site. A comparison of current (2017–2019) and historical (1956–1957) temperature data in touristically poorly visited Predjama Cave shows lower increase as in PJ and is completely related to outside cave conditions. In the case of Škocjanske Jame (Tiha Jama), air temperature has not significantly increased since the historical 1928 measurements because the monitoring site looks to be morphologically isolated from significant impacts of outside climate and visitors.

**Supplementary Information:**

The online version contains supplementary material available at 10.1007/s42452-021-04881-1.

## Introduction

Recent years have seen numerous studies related to environmental issues in show caves [1–4],etc.). Many of them show an increase of tourist visits from year to year. In more and more caves, the impact of visitors on the fragile cave environment is the subject of systematic microclimatic and biological monitoring [5, 6]. There are more than 14,000 registered caves in Slovenia (Cave Register [7], the country of the Classical Karst, but only 22 of them are open as show caves. Two show caves are among the top ten top tourist destinations in Slovenia. These are Postojnska Jama, with 870,000 visitors in 2019, and the Škocjanske Jame, a UNESCO heritage site, which received 190,000 visitors in 2019. As a result of the COVID-19 pandemic, visitor numbers in Slovenia’s show caves were down by around 80% in 2020 and similar is expected for 2021.

The first cave studied for this research is the massively visited Postojnska Jama, the second is the little-visited Predjama Cave (< 10,000 visitors in 2019), and the third is the strongly visited Škocjanske Jame. Microclimatic monitoring is carried out at multiple locations in all three caves [4, 8–10] and historical data on cave meteorology are available [11–14].

The karst area of SW Slovenia is also the site of Vilenica, the oldest show cave in Europe, where tourist activity has been recorded since 1633 [15]. Show caves in Slovenia are the subject of systematic studies related to understanding the impact of visitors on caves [9]. Short-lived increases in air temperatures of up to 0.5 °C are reported from the most visited parts of Postojnska Jama [6, 8]. Air flows into this cave from the exterior through the main entrances in response to forced convection [6]. The artificial opening of the main entrance in 1866 is an additional factor which helps ensure that Postojnska Jama is well ventilated deep inside [8, 10]. In this sense, changes in the outside cave climate have an impact on the cave climate [16].

Microclimatological studies are organised in show caves worldwide in order to monitor the impact of tourism on cave climate. The difference between the mean exterior temperature (17.86 °C) and mean air temperature in the Nerja Caves in Spain (19.48 °C) is 1.62 °C [1]. In a study by Liñán et al. [3] for the Nerja-Pintada cave system, the mean exterior temperature values are 18.97 °C, the section of the Nerja Caves open to tourists has mean temperature values of 17.41 °C, 19.08 °C and 19.02 °C, and the section not open to tourists has mean temperature values of 19.46 °C, 19.98 °C and 19.86 °C. This emphasises the differences between mean exterior temperatures and cave temperatures.

Lascaux Cave, which is not open to visitors, is not overheated with respect to the outside climate. In the period February 2015–February 2016, the average exterior temperature was 12.94 °C, while the average air temperature in the cave was 12.6 °C [2].

There are also caves, which are much warmer than surface conditions. The air temperature in the Kateřinska show cave in the Czech Republic increases in a direction from the cave entrance to the interior of the cave. Average outside air temperature for the period 2010–2012 was 5.91 °C, while more distant sections of the cave showed temperatures of 7.43 °C, 7.62 °C and 8.05 °C [17].

Geothermal heating is the most probable reason why temperatures inside the Kartchner Caverns (Arizona, USA) are 1.7°–4 °C higher than the mean surface temperature [18]. Be that as it may, anthropogenically induced changes cannot be excluded, because the changes represent a combination of anthropogenic and regional natural causes [19].

The air temperature at Carlsbad Caverns (USA) is not only heated up several times a day during guided tours, but is also constantly held at a higher level all year round. In the King’s Palace and the Big Room, the two most visited sections convective ventilation and the influence of barometric ventilation are almost completely overwhelmed by the anthropogenic influence [20].

We have evidence of temperature changes in different environments, not only in karst caves. European summer temperatures in the period 1986–2015 were anomalously high (1.3 °C higher) and there is no evidence of any period in the last 2000 years being as warm [21]. Additionally, the climatic evolution of the Alpine basin in the period 1950–2012 caused a pronounced glacial decline and an increase in air temperatures (+ 1.8 °C) [22].

Analyses of temporal trends (1961–2004) for surface air temperature on the Yunnan Plateau (SW China), which includes karst areas showed that warming trends of night-time minimum temperature are more pronounced than those of day-time maximum temperature, especially during the winter season, whereas summer warming is mostly confined to the southern part of the Yunnan Plateau [23].

Karst-related C sink could respond to future climate change quickly and needs to be considered in the modern carbon cycle model. SW China has experienced dramatic change in regional climate as regards total karst-related C sink (TSC). Between 1970 and 2010, TCS decreased by about 19% as a result of an increase in air temperature (from 0.33 °C to 1.04 °C) and a decrease in precipitation (from 156 to 106 mm) [24].

Future climate estimates give different scenarios. One estimate for the year 2050 found that 77% of future cities tend to shift towards warmer conditions, while cities from the tropics will shift to drier conditions [25].

Other causes of climate changes are discussed alongside industrial evolution, including solar forcing, planetary motion and volcanic activity [26]. Scafetta [27] showed that global surface temperature records since 1850 present fluctuations (including a large ~ 60-year oscillation) that cross-correlate well with measurable astronomic oscillations, which are linked to planetary motion.

Variations in the flow of the Atlantic Meridional Overturning Circulation may be responsible for some of the 2–15 year variability observed in global land temperatures. Strong inter-annual and decadal variations observed in the average land surface temperature records represent a true climate phenomenon, not only during the years when fluctuations on the timescale of 2–15 years had been previously identified with El Niño events [28].

Whether observed climate changes are due to human activity and/or are part of natural systems fluctuations remains a major stumbling block to effective adaptation action and risk management [29].

The aim of this research is to use published historical air temperature data [11–14] to determine possible changes in air temperatures measured in the show caves of Slovenia in the last years (2015–2019). By comparing cave air temperatures with outside air temperatures and cave visitor numbers, we intended to verify possible influence of natural and/or anthropogenic causes on cave temperature changes. Based on the rich history of karst research in Slovenia [30, 31], including historical meteorological data sets, it would be possible to provide a reliable record of show cave temperature changes over the last 80–90 years.

## Data and methodology

**Fig. 1:**
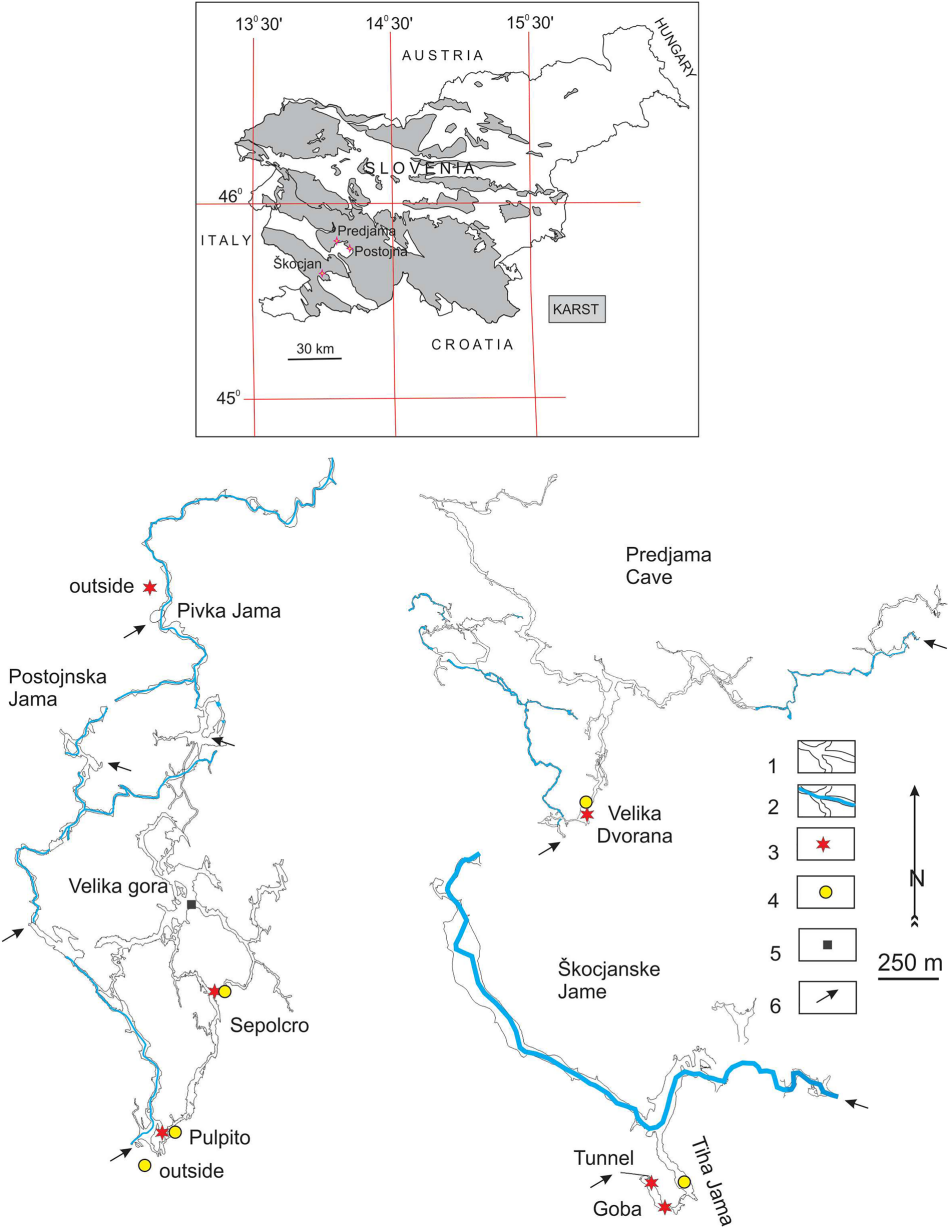
Cave ground-plan maps with historical and current air temperature monitoring sites. 1—dry cave passages, 2—water cave passages, 3—current air temperature monitoring site, 4—historical air temperature monitoring site [11–14], 5—historical air temperature monitoring site [32], 6—cave entrances

The current air temperature measurements presented within this study were collected at five monitoring sites from three show caves in Slovenia (Fig. 1). An additional temperature monitoring site is located outside the caves in the forest near the entrance to Pivka Jama (part of the Postojnska Jama cave system) without additional human impact on the monitoring site. Hourly measurements were taken by Baro-Diver data loggers (Van Essen) with accuracy ± 0.1 °C and resolution 0.01 °C with regular calibration based on the new calibrated instruments that replaced older ones.

To compare historical air temperature measurements with the present data set, two historical monitoring sites in Postojnska Jama [12] have been re-established. The Pulpito site (by the speleothem known as the Pulpit) was re-activated in 2017, while the Sepolcro site (by the speleothem known as the Sepulchre or Baldachin) was re-activated in 2015. The Velika Dvorana chamber in Predjama Cave, which was also a historical temperature monitoring site [11, 13], has been collecting hourly temperature data since 2009 [9]. Mean monthly values were calculated for historical and modern air temperature measurements. For Postojnska Jama, available and published historical mean monthly air temperatures from October 1933 to December 1937 [12] were compared with mean monthly air temperature values for the Pulpito monitoring site for the period 2017–2019. Historical data from the Sepolcro monitoring site [12], which are available for 1935–1937, were compared with current mean monthly values for the period 2015–2019. In the case of Velika Dvorana in Predjama, historical mean monthly air temperature values from February 1956 to February 1957 [13] were compared with mean monthly air temperature values for the period 2017–2019.

The section of the Škocjanske Jame known as Tiha Jama (“Quiet Cave”) has been subject to regular microclimatic monitoring since December 2017. Modern air temperature data from the Škocjanske Jame were compared with historical measurements taken from occasional measurements in 1928 [14]. Historical and modern air temperature measurements do not represent the same site but are about 150 m away (Fig. 1).

Outside air temperature measurements near the entrance to Pivka Jama were compared with historical surface measurements in front of Postojnska Jama (Fig. 1) and also for Predjama Cave, since the latter is expected to have similar climatic conditions. The distance between Postojnska Jama and Predjama Cave is about 15 km as the crow flies.

We are aware that historical air temperature measurements [11–14] were not as precise as current hourly measurements. To get the best comparability between old and modern data sets, mean monthly temperature values were used for calculations. It should nevertheless be borne in mind that the historical data have precision of ± 0.25 °C what shows some limits for evaluation. We need to know that temperature oscillations during one-year period in the cave (Pulpito site in Postojnska Jama) can be < 1.0ºC [8], so the historical instrument must have been precise enough for detection such small oscillations.

Pearson correlation coefficients (PCCs) are calculated to see the relationships between mean annual cave air temperature measurements, mean annual outside air temperatures and number of visitors. PCC has a value between + 1 and -1, where 1 is total positive linear correlation, 0 is no linear correlation, and -1 is total negative linear correlation. Annual visitor numbers have been obtained from cave management.

*T* test was used to determine if there is a significant difference between the historical mean monthly air temperatures and current values for Postojnska Jama locations because those sites have the most extensive historical data available.

## Results and discussion

### Postojnska Jama air temperature

**Fig. 2:**
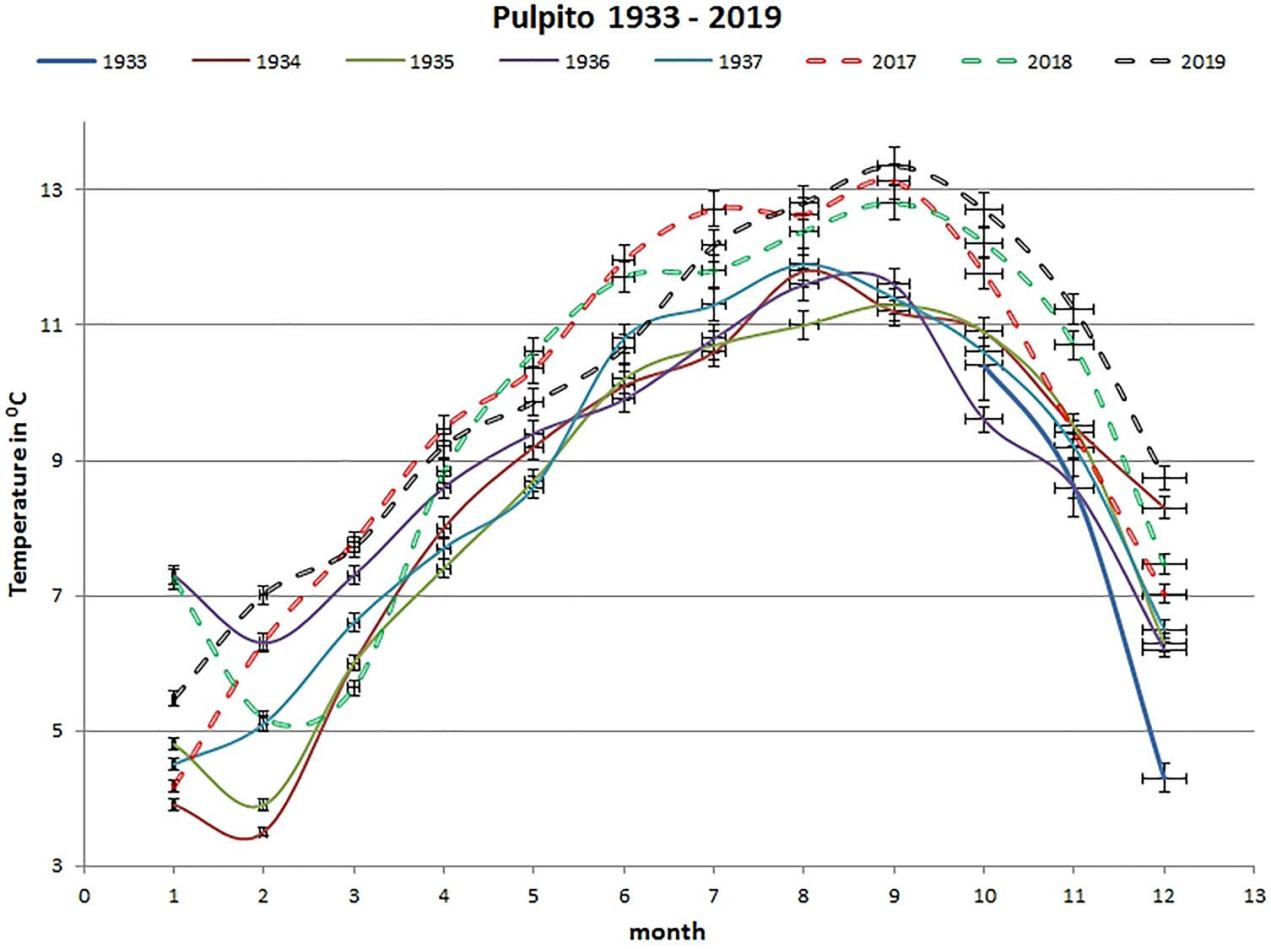
Comparison of mean monthly historical [12] and current air temperatures (in °C) at the Pulpito monitoring site in Postojnska Jama, error bars show 2% standard error

A historical air temperature data set covering a period of four years and three months (October 1933 to December 1937) at the Pulpito site (Figs. 1 and 2) was compared with recent mean monthly air temperatures over the course of three years (2017–2019). Historical and current temperature measurements took place at the same locations. Mean monthly cave air temperature values in the more recent three-year period are higher than the historical measurements [12]. The consistent difference between the current and historical measurements is even more evident if we calculate t test based on mean monthly air temperature values between historical (1934–1937) period and recent (2017–2019) period (Table 1). When the probability value (p) is < 0.05 the difference between historical and current mean monthly air temperatures is statistically significant. If p > 0.05 the difference is insignificant. The most significant temperature difference is for the months April–November (Table 1). Colder months (December–March) are statistically insignificant if we compare historical (1934–1937) air temperatures with current measurements (2017–2019). Air temperature monitoring at the Pulpito site, which is located only around 150 m inside the Postojnska Jama, shows that over the course of 82 years (1937–2019) the higher temperature rises are detected in warmer part of the year, while colder part of the year does not show significant differences between historical and current data.

**Table 1:** *T* test calculated for mean monthly temperatures (historical 1934–1937 versus current 2017–2019) for Postojnska Jama (PJ) sites (Pulpito, Sepolcro) and outside

	Sepolcro PJ (historical versus current)	Pulpito PJ (historical versus current)	Outside (historical versus current)
	*p*	*p*	*p*
January	**0.0000000238**	0.682688	0.73947
February	**0.000035**	0.15116	0.954542
Marec	**0.00008**	0.451045	0.986
April	**0.000007**	**0.014676**	0.377343
May	**0.000003**	**0.006798**	0.604005
June	**0.000004**	**0.031754**	0.366802
July	**0.000002**	**0.004791**	**0.018774**
August	**0.000001**	**0.010295**	**0.000719**
September	**0.000001**	**0.000148**	0.483935
October	**0,0,000,003**	**0.003436**	0.442822
November	**0.00000043**	**0.029111**	0.069875
December	**0.000001**	0.175536	0.584555

When probability *p* < 0.05 the difference between historical and current temperatures is statistically significant (bold numbers), when *p* > 0.05 the difference between historical and current temperatures is statistically insignificant

**Fig. 3:**
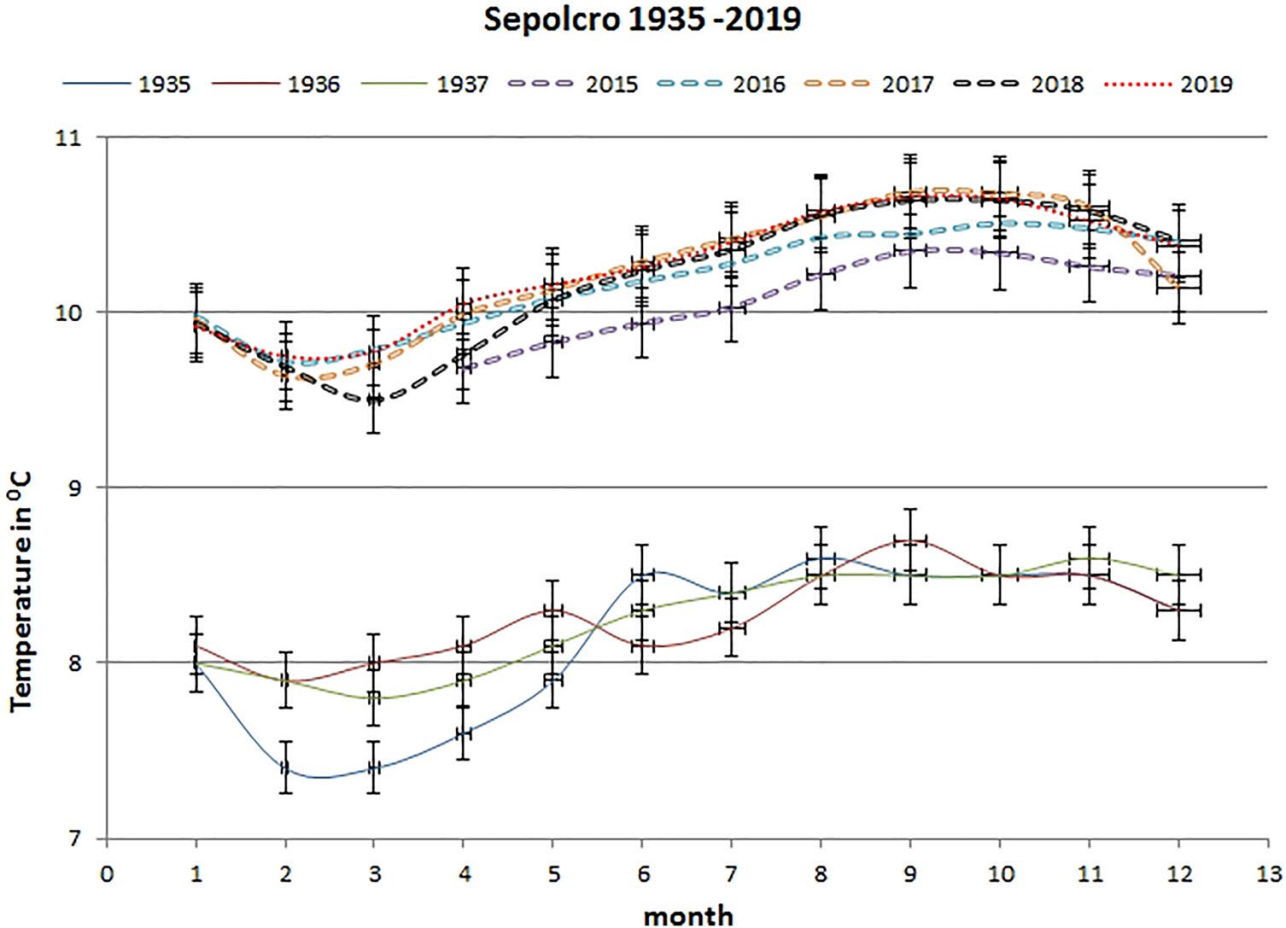
Comparison of mean monthly historical [12] and current air temperatures (in °C) at the Sepolcro monitoring site in Postojnska Jama, error bars show 2% standard error

**Fig. 4:**
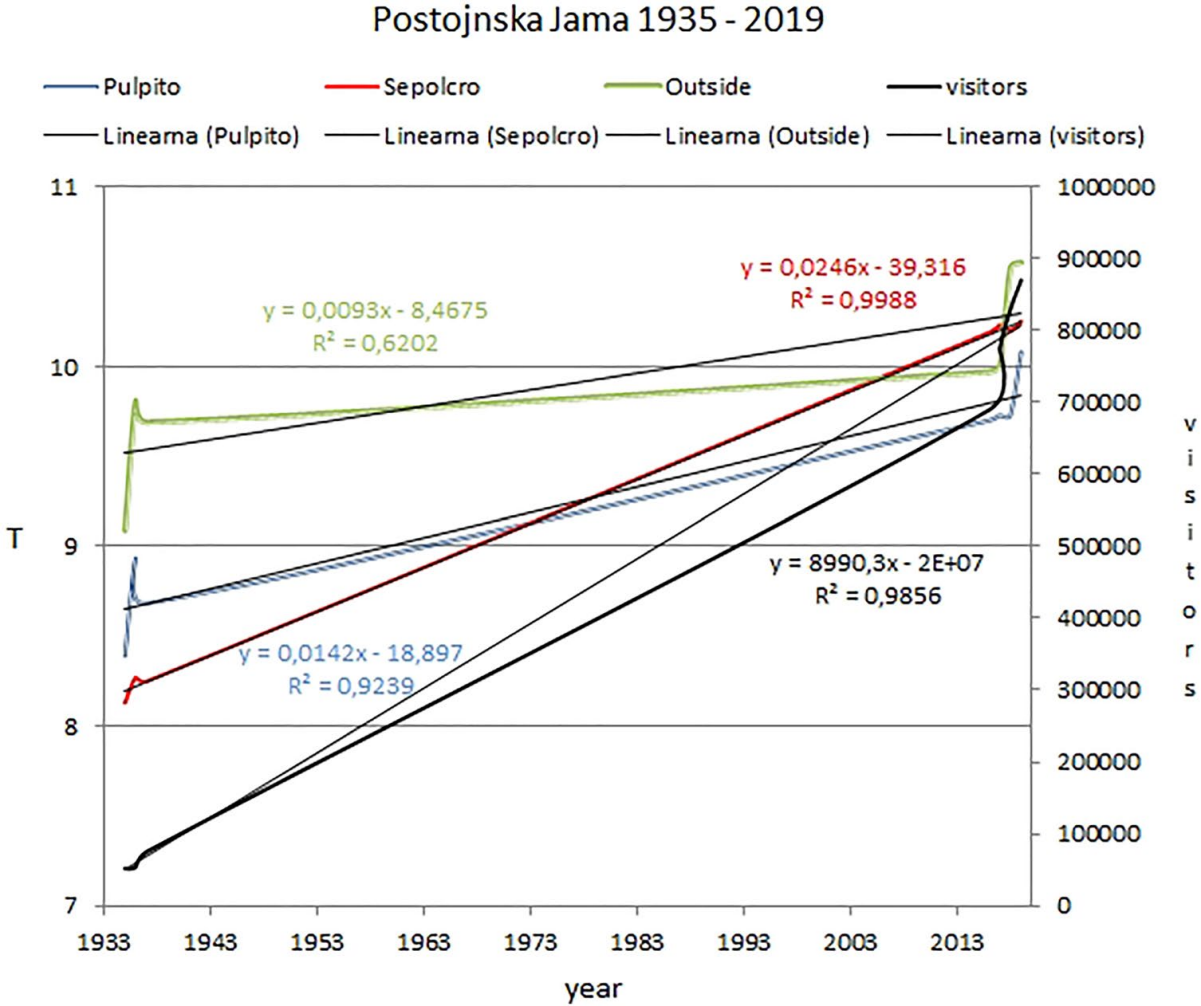
Mean annual air temperatures (in °C) in Postojnska Jama and outside the cave with annual visitor numbers for the period 1935–2019. Historical data are from Crestani and Anelli [12]

The second Postojnska Jama temperature monitoring site, Sepolcro (Fig. 1), is situated about 800 m inside the cave. At this location too, mean monthly air temperatures are clearly higher in the period from April 2015 to December 2019 than they were in the period 1935–1937 (Fig. 3). The Sepolcro site shows a higher air temperature rise (Fig. 4) than the Pulpito site (Fig. 2).

Mean monthly temperature values (Fig. 3) and the difference between the current (2016–2019) and historical (1935–1937) periods at the Sepolcro site show constantly higher values in the modern period than in the past. T test confirms that historical versus current mean monthly temperatures are significantly different with *p* < 0.05 during the whole-year period (Table 1).

In September 1852, Schmidl [32] measured the average daily air temperature at floor level of the Velika Gora chamber in Postojnska Jama 8.5 °C (Fig. 1). Comparing this historical measurement with recent measurements, it is apparent that current air temperature is almost 2 °C higher than it was in 1852 [6]. This is comparable to the Sepolcro site temperature rise (Fig. 3). For the period 2015–2017 [4], the Sepolcro site showed temperatures that were 1.74°–2.64 °C higher than in 1933–1937 [12]. Regarding the difference between the mean monthly historical (1935–1937) and current (2016–2019) temperature values (Table 2), the current air temperature at Sepolcro site is 2 °C higher as it was in the past.

**Table 2:** Comparison between mean air temperatures (in °C) for historical and modern periods for Postojnska Jama (PJ) sites (Pulpito, Sepolcro), Predjama site (VD—Velika Dvorana) and outside

Monitoring site	Period	Mean air T (ºC)
PJ Pulpito	1934–1937	8.64
PJ Pulpito	2017–2019	9.83
PJ Sepolcro	1935–1937	8.21
PJ Sepolcro	2016–2019	10.21
Predjama—VD	1956–1957	6.83
Predjama—VD	2017–2019	7.29
Outside	1934–1937	9.72
Outside	2015–2019	10.27

### Visitors and cave air temperature in Postojnska Jama

Pearson correlation coefficients (PCCs) calculated on the basis of mean annual air temperatures and number of visitors per year for the period 1935–2019 (Table 3 and Fig. 4) suggest that the air temperature curve at the Sepolcro monitoring site and the visitor number curve have a very strong correlation (PCC = 0.99). The air temperature curve at the Pulpito site and the visitor number curve (Fig. 4) also show a strong correlation (PCC = 0.96). Outside cave air temperature and Pulpito air temperature have a PCC of 0.88, which is a strong correlation but lower than those of the cave sites, while Sepolcro air temperature and outside air temperature have a PCC of 0.78, which is the lowest correlation coefficient calculated in Table 3.

**Table 3:** Pearson correlation coefficients (PCCs) for Postojnska Jama (1934 –2019) and Predjama Cave (1942–2019) calculated on the basis of mean annual air temperature values and annual visitor numbers

	Outside	Sepolcro	Pulpito
Outside	–	0.78	0.88
Postojna Cave (PJ) visitors	0.81	0.99	0.96
PJ Sepolcro	0.78	–	0.96
Predjama—Velika Dvorana	0.80	–	–

0.90–0.99 (very strong correlation)

0.70–0.89 (strong correlation)

0.40–0.69 (medium correlation)

Regarding the increased trends (Fig. 4), *R*^2^ (multiple regression) is lowest for outside temperature (*R*^2^ = 0.62) and highest for Sepolcro air temperature (*R*^2^ = 0.99). For visitors (*R*^2^ = 0.98) and the Pulpito site (*R*^2^ = 0.92), increased trends are high. Outside air temperature increase in the calculated period 1935–2019 is lower than for the cave sites (Sepolcro and Pulpito). A comparison of annual visitor numbers and mean annual air temperatures in at Postojnska Jama for the period 1935–2019 shows increasing trends (Fig. 4). Mean annual air temperature at the Sepolcro site and the annual visitor numbers show similar increasing trends (Fig. 4).

### Predjama cave

In recent years, Predjama Cave has received fewer than 10,000 visitors per year [9]. Compared to the heavily visited Postojnska Jama, Predjama Cave is poorly visited. In this sense, it offers an ideal opportunity to see changes in air temperature by comparing historical data [11, 13] and current data from which a strong human impact due to visitors can be excluded.

**Fig. 5:**
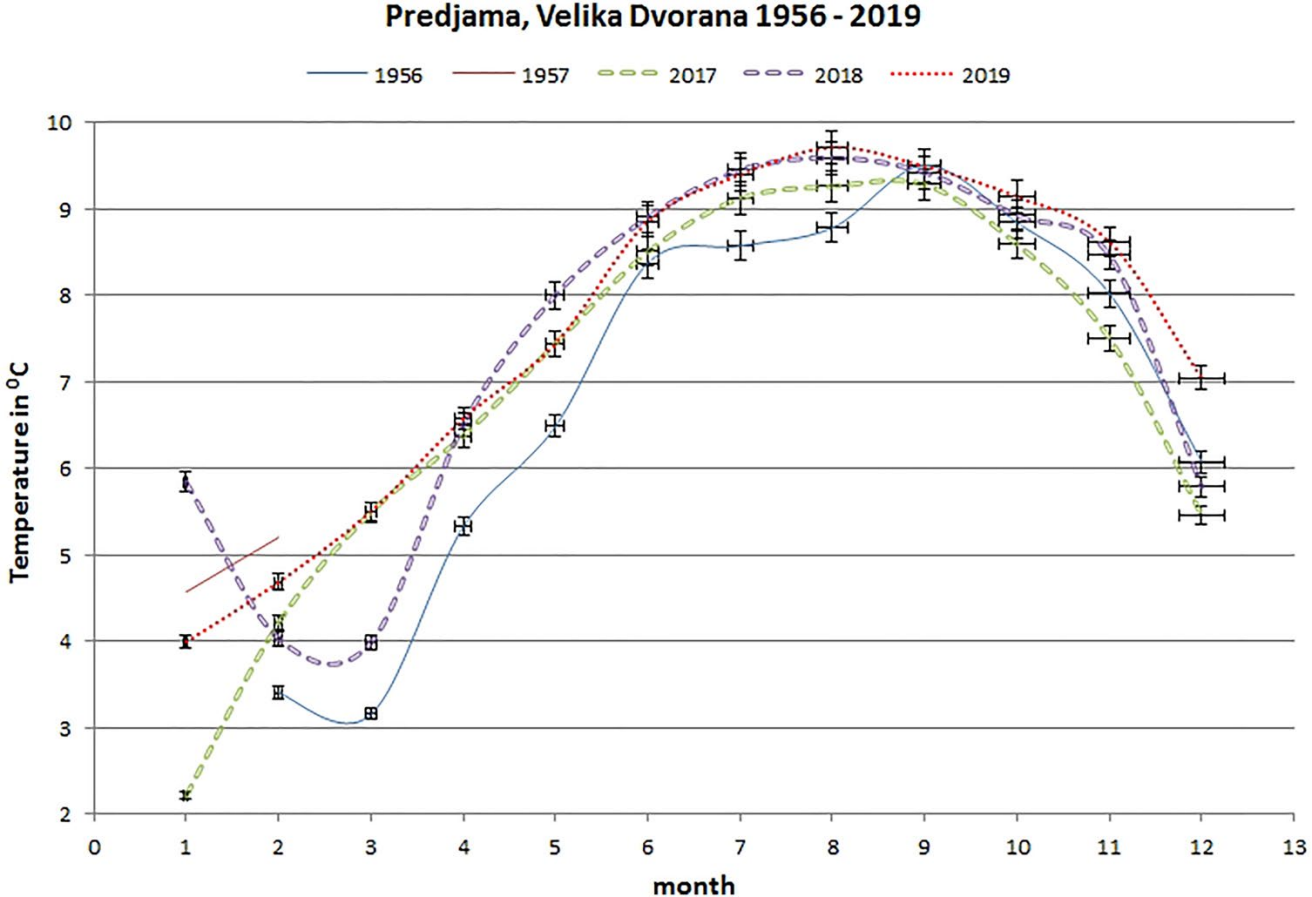
Comparison of mean monthly historical [13] and current air temperatures (in °C) at Velika Dvorana in Predjama Cave, error bars show 2% standard error

Predjama Cave does not have such a long historical temperature data set as Postojnska Jama, but measurements of mean monthly temperature values in the Velika Dvorana collapse chamber over a period of at least a year (February 1956 to February 1957), as published by Habe [13], provide some information (Fig. 1). The historical data set was compared to current measurements (2017–2019) from the same cave room (Figs. 1 and 5). We can see that present-day air temperature measurements are higher than historical measurements from February to August. The difference between current and historical temperatures is not so obvious in the period from September to December. The mean January temperature in the period 2017–2019 was even lower than it was in January 1957. The highest temperature difference (1.84 °C) between modern and historical data is recorded in March, where the current data show higher temperatures. Historical September values are higher than modern measurements and show a negative temperature difference (−0.56 °C). The last three months of the year (October–December) show only slightly higher temperatures in modern measurements compared to historical records.

**Fig. 6:**
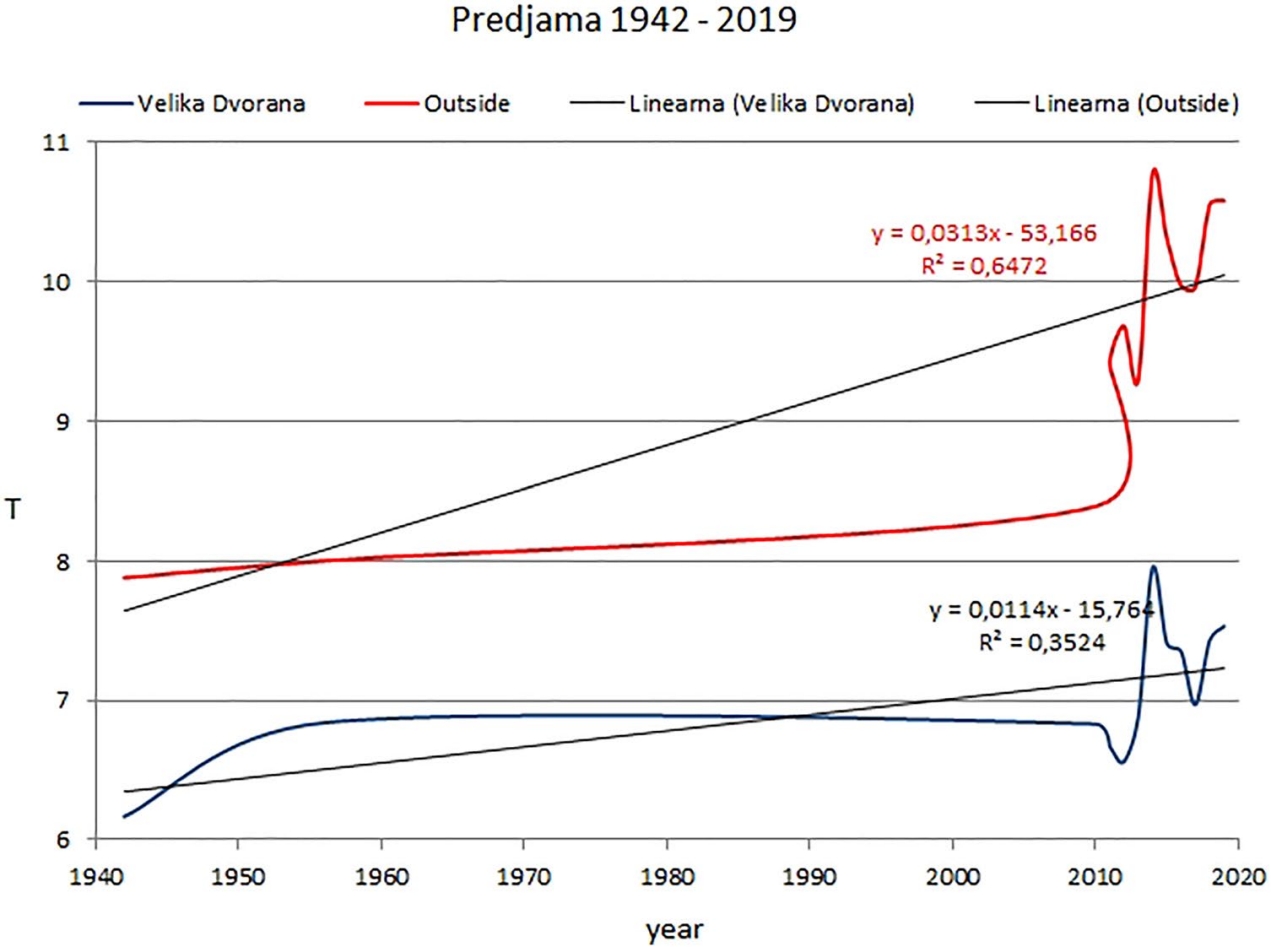
Mean annual air temperature (in °C) in Velika Dvorana (Predjama Cave) and outside the cave for the period 1942–2019. Historical data are from [11, 13]

Mean annual air temperature in the Velika Dvorana chamber measured by Anelli (1941–1944) from December 1942 to August 1943 and the measurements performed by Habe [13] in the period 1956–1957 are presented on Fig. 6. We can see temperature increase in the period 1942–2019 for Predjama Cave and outside cave location. The highest increases in outside air temperature relate to recent periods 2010–2012, 2013–2014 and 2016–2019.

The temperature rise in the period 1942–2019 is greater outside the cave (*R*^2^ = 0.64) than it is in the Velika Dvorana chamber inside Predjama Cave (*R*^2^ = 0.35). The temperature increase in Predjama Cave is lower than at the monitoring sites in Postojnska Jama (Pulpito and Sepolcro sites, Table 2) because there is no significant heat from visitors.

### Outside air temperature

Historical outside air temperature data were taken from Crestani and Anelli [12]. For Postojna area, other historical data do not exist. An outside air temperature monitoring site was set up in front of the main entrance to Postojnska Jama (Fig. 1) and protected by a wooden structure. Mean monthly temperature values are available from October 1933 to December 1937 [12]. Current hourly temperature measurements were taken on the surface in the forest above the Postojnska Jama near the entrance to Pivka Jama. The distance between the historical and modern outside air temperature monitoring sites is about 2.7 km as the crow flies.

A study [6] noted that a comparison of outside air temperatures for 1933–1937 with those for 2009–2013 could be considered unreliable because the average air temperature in 1933–1937 was higher than in 2009–2013. This was explained by the fact that the site of the historical measurements was located on the south-facing slope in front of the cave, where air temperatures can be elevated [6]. A comparison of the historical period 1934–1937 with 2015–2019 on the basis of mean monthly temperatures (Table 2) shows that outside air temperature is 0.55 °C higher in current period than it was in 1934–1937. T test (Table 1) calculated from mean monthly temperature values between historical and current temperatures shows that only July and August (1933–1937) are significantly lower as modern period (2015–2019) temperatures. All other months do not show that modern temperatures are higher as historical.

Mean outdoor air temperature in Slovenia increased by 1.7 °C between 1961 and 2011 (http://meteo.arso.gov.si/uploads/probase/www/climate/text/en/publications/PSSbrosura_spread_ENG.pdf). This might help understand why historical air temperatures outside Postojnska Jama from October 1933 to December 1937 [12] cannot be directly compared with modern data because of the different location and because of the lack of official historical meteorological station in the area of Postojna.

### Škocjanske Jame

In the case of the Škocjanske Jame (Tiha Jama, Fig. 1), we started measuring air temperature at hourly intervals at the Tunnel site on 19 December 2017 and at the Goba site on 5 February 2018. Mean annual air temperature at the Goba site (Table 4) was 12.11 °C in 2019 and is comparable to the outside mean annual air temperature, which was 12.08 °C in 2019 (https://meteo.arso.gov.si/met/sl/).

**Table 4:** Mean annual air temperature in the Škocjanske Jame and outside in °C

	2018	2019
Tunnel	12.29	12.5
Goba	–	12.11
Outside	12.12	12.08

Kranjc and Opara [33] determined an air temperature range of 11–12.5 °C (with occasional peaks of up to 13.5 °C) for the period May 1997–May 1999 in Tiha Jama (at the Goba site). Vercelli [14] recorded values of 11.5°–13 °C for air temperature in Tiha Jama (Fig. 1) in the period 8 January–23 December 1928. He collected 15 measurements with a mean value of 12.28 °C. This was the period before the man-made tunnel to Tiha Jama was completed (the tunnel opened in 1933). We can see that a comparison of sporadic air temperature measurements in 1928 [14] with recent measurements (Table 4) does not show a significant difference in cave temperature over 92 years. In the case of the Škocjanske Jame (Tiha Jama), air temperature has not increased significantly since the historical measurements, as is the case in of Postojnska Jama and Predjama Cave. The reason is probably the morphology of the cave passages with monitoring site locations and the underground watercourse that governs air flows between the cave and the exterior and also not as high visitor numbers as in the Postojnska Jama. Anyway, we need to have in mind that historical data represent only limited number of measurements.

### Difference between historical and current air temperature measurements

When comparing the Postojnska Jama monitoring sites (Pulpito and Sepolcro, Table 1), we can see that Pulpito shows the highest temperature increase in comparison with the historical data [12] in summer months and the lowest temperature increase in winter months. Sepolcro site reflects whole-year temperature rise in the modern period comparing to historical period. The increased temperatures in recent period can be explained by the temperature behaviour in the cave in respect of outside conditions. Increasing number of visitors to Postojnska Jama represent additional heat input to cave environment. It was confirmed that Postojnska Jama temperatures in the period 2010–2020 and also outside cave are increasing [31].

Predjama Cave, on the other hand, shows 0.46 ºC of modern temperature increase in comparison with the historical data (Table 2) regarding the mean annual values. In Predjama Cave, we can see only natural warming due to the outside heating. Because of the small number of visitors, additional temperature increase due to visitors must be excluded.

In Postojnska Jama and in Predjama, all historical mean annual air temperature data are lower as modern (Table 4). The temperature increase in Predjama Cave in modern period is attributed only to outside temperature, while temperature increase in Postojnska Jama depends not only on outside temperature increase but also on the number of visitors (Table 3, Fig. 4).

In Škocjanske Jame, air temperature has not increased significantly in comparison with historical measurements, whereas in Postojnska Jama and Predjama Cave it has increased. It looks that Tiha Jama monitoring site in Škocjanske Jame represents morphologically stable environment where outside heating and visitor numbers do not significantly disturb cave climate conditions. With strongly increased visitors, the situation might change.

## Conclusions

Mean monthly air temperature data sets from modern monitoring periods calculated from hourly measurements were compared with mean monthly historical data sets [11–14] in the case of three show caves in Slovenia. Historical and modern temperature measurements were taken at the same locations, except in the case of the Škocjanske Jame where the monitoring sites are ~ 150 m apart.

Air temperature monitoring at the Pulpito site, which is situated near the entrance to Postojnska Jama, shows that cave air temperature has increased over the course of 82 years (1937–2019). Recent temperatures are significantly higher as historical during warmer part of the year (April–November) while colder part of the year is not significantly different (Table 1). The mean annual temperature difference between the 2017–2019 and the 1934–1937 period for the Pulpito site stands at + 1.19 °C in advantage to modern period (Fig. 2).

The second Postojnska Jama monitoring site, known as Sepolcro (Fig. 1), is situated about 800 m inside the cave and shows noticeably higher mean monthly temperatures in the period April 2015–December 2019 than in the period 1935–1937 (Fig. 3). The Sepolcro site shows a higher increase in air temperature than the Pulpito site. Comparing modern temperatures with historical, there is a year-round significant temperature difference at Pulpito. Mean annual temperature for the period 2016–2019 at Pulpito site is + 2.00 °C higher than in historical time (1935–1937) (Table 2).

Pearson correlation coefficients (PCCs) calculated on the basis of mean annual air temperatures and annual visitor numbers for the period 1935–2019 (Table 3) suggest a very strong correlation (PCC = 0.99) between the Sepolcro monitoring site and visitor numbers, while there is also a strong correlation (PCC = 0.96) between the Pulpito site and visitor numbers. Outside air temperature and air temperature at the Pulpito site likewise give a strong correlation (PCC = 0.88), while there is a weaker correlation (PCC = 0.78) between the Sepolcro site and outside air temperature, what can be explained with the fact that Sepolcro is about 800 m deep inside the cave and more distant from impact of outside conditions.

In the case of poorly visited Predjama Cave, the historical one-year long period February 1956–February 1957 [13] was compared to the period 2017–2019. The temperature historical data for Predjama Cave are not as reliable as for Postojnska Jama. The mean annual difference for Predjama Cave shows that air temperatures in the period 2017–2019 are + 0.46 °C higher than they were in the period February 1956–February 1957 (Table 2).

Comparing the historical period 1934–1937 with the period 2015–2019 in terms of mean annual outside temperatures (Table 2), the measurements in the modern period are + 0.55 °C higher than in 1934–1937. Historical outside air temperature measurements have not been located on the same site as that of the modern ones and have received much more heat as if they were placed in forest. Historical outside temperatures measured in front of the Postojnska Jama must be treated with some caution.

A comparison of 15 historical air temperature measurements taken in the Škocjanske Jame (Tiha Jama) in 1928 [14] with recent measurements (Table 4) reveals no significant difference in cave temperature over the course of 92 years (1928–2019). In the case of Tiha Jama, air temperature has not increased significantly in comparison with historical measurements, as is the case in Postojnska Jama and Predjama Cave. This is probably related to the position of monitoring site, which looks to be morphologically preserved from significant impacts of outside climate and visitors. Only limited number of historical measurements at Škocjanske Jame can also represent the lack for sufficient temperature comparability between historical and modern times.

The increase in outside air temperature in the period 1934–2019 is smaller than the increase in air temperature in the massively visited Postojnska Jama (Sepolcro site) over the same period (Table 2). The temperature increase in Postojnska Jama proves that visitors have represented an additional factor in cave air temperature increases over the last 85 years, especially for Sepolcro site.

The temperature increase in the little-visited Predjama Cave is smaller than the increase in outside temperature in the period 1956–2019, where the temperature increase in Predjama Cave must be attributed to the increase in outside temperature.

Historical temperature data sets from show caves are valuable information even if they are not as reliable as modern measurements. Such old published measurements are very rare and can with some limits help to understand cave micro-climate changes.

## Supplementary Information

Below is the link to the electronic supplementary material.

Supplementary file1 (DOCX 15 KB)

Supplementary file2 (DOCX 19 KB)

Supplementary file3 (DOCX 16 KB)

Supplementary file4 (DOCX 14 KB)

Supplementary file5 (DOCX 16 KB)

Supplementary file6 (DOCX 14 KB)

Supplementary file7 (DOCX 53 KB)
