# Supplementary material for: Comparison of historical and current temperatures in show caves (Slovenia)
Source: SN Appl Sci. 2021 Dec 4;4(1):1. doi: 10.1007/s42452-021-04881-1 (PMC8643192; doi:10.1007/s42452-021-04881-1)
Supplement: Supplementary file 1 — Supplementary file1 (DOCX 15 KB) [file 42452_2021_4881_MOESM1_ESM.docx]

- Correlation analyses between historical and modern air *T* in 3 show caves is discussed.
- Actual air *T* are higher than historical data in two show caves.
- Outside cave warming and visitors are causing rise of cave *T* over historical period.
