# Supplementary material for: Comparison of historical and current temperatures in show caves (Slovenia)
Source: SN Appl Sci. 2021 Dec 4;4(1):1. doi: 10.1007/s42452-021-04881-1 (PMC8643192; doi:10.1007/s42452-021-04881-1)
Supplement: Supplementary file 2 — Supplementary file2 (DOCX 19 KB) [file 42452_2021_4881_MOESM2_ESM.docx]

Fig. 1 Cave ground-plan maps with historical and current air temperature monitoring sites. 1 – dry cave passages, 2 –water cave passages, 3 – current air temperature monitoring site, 4 – historical air temperature monitoring site (Vercelli 1931, Crestani and Anelli 1939, Anelli 1941–1944, Habe 1970), 5 – historical air temperature monitoring site (Schmidl 1854), 6 – cave entrances.

Fig. 2 Comparison of mean monthly historical (Crestani and Anelli 1939) and current air temperatures (in °C) at the Pulpito monitoring site in Postojnska Jama, error bars show 2% standard error.

Fig. 3 Comparison of mean monthly historical (Crestani and Anelli 1939) and current air temperatures (in °C) at the Sepolcro monitoring site in Postojnska Jama, error bars show 2% standard error.

Fig 4. Mean annual air temperatures (in °C) in Postojnska Jama and outside the cave with annual visitor numbers for the period 1935–2019. Historical data are from Crestani and Anelli (1939).

Fig. 5 Comparison of mean monthly historical (Habe 1970) and current air temperatures (in °C) at Velika Dvorana in Predjama Cave, error bars show 2% standard error.

Fig. 6. Mean annual air temperature (in °C) in Velika Dvorana (Predjama Cave) and outside the cave for the period 1942–2019. Historical data are from Anelli (1941–1944) and Habe (1970).

Table 1 T-test calculated for mean monthly temperatures (historical 1934–1937 versus current 2017–2019) for Postojnska Jama (PJ) sites (Pulpito, Sepolcro) and outside. When probability p < 0.05 the difference between historical versus current temperatures is statistically significant (bold numbers), when p > 0.05 the difference between historical versus current temperatures is statistically insignificant.

Table 2 Comparison between mean air temperatures (in °C) for historical and modern periods for Postojnska Jama (PJ) sites (Pulpito, Sepolcro), Predjama site (VD - Velika Dvorana) and outside.

Table 3 Pearson correlation coefficients (PCCs) for Postojnska Jama (1934 –2019) and Predjama Cave (1942–2019) calculated on the basis of mean annual air temperature values and annual visitor numbers.

Table 4 Mean annual air temperature in the Škocjanske Jame and outside in °C.
