# Supplementary material for: Comparison of historical and current temperatures in show caves (Slovenia)
Source: SN Appl Sci. 2021 Dec 4;4(1):1. doi: 10.1007/s42452-021-04881-1 (PMC8643192; doi:10.1007/s42452-021-04881-1)
Supplement: Supplementary file 4 — Supplementary file4 (DOCX 14 KB) [file 42452_2021_4881_MOESM4_ESM.docx]

**Cover letter**

Manuscript title: **Comparison of historical and current temperatures in show caves (Slovenia)**

Corresponding author: Stanka Šebela*

The manuscript deals with correlation analyses between historical and modern air temperatures in three show caves in Slovenia. It was shown that actual air temperatures are higher than historical data in two show caves. The cause is general outside cave warming and increase of visitors. It was confirmed that generally stable underground air temperatures can be impacted with human presence and outside cave warming that is transmitted to the cave over historical time period.

Stanka Šebela
