# Supplementary material for: Comparison of historical and current temperatures in show caves (Slovenia)
Source: SN Appl Sci. 2021 Dec 4;4(1):1. doi: 10.1007/s42452-021-04881-1 (PMC8643192; doi:10.1007/s42452-021-04881-1)
Supplement: Supplementary file 5 — Supplementary file5 (DOCX 16 KB) [file 42452_2021_4881_MOESM5_ESM.docx]

**Title page**

Manuscript title: **Comparison of historical and current temperatures in show caves (Slovenia)**

Corresponding author: Stanka Šebela*

*ZRC SAZU IZRK, Titov trg 2, 6230 Postojna, Slovenia, [sebela@zrc-sazu.si](mailto:sebela@zrc-sazu.si)

**Acknowledgements**

Study was performed within the programme *Karst Research* (P6-0119) financed by Slovenian Research Agency, research project *Karst Research for Sustainable Use of Škocjan Caves as World Heritage* (L7-8268) co-financed by Slovenian Research Agency and Park Škocjanske Jame, research project *Methodology for monitoring the sustainable use of karst show caves with automatic measurements – role model – Postojna cave* (L6-9397) co-financed by Slovenian Research Agency, Postojnska jama d.d. and REODOM d.o.o. and by the project operation “Development of research infrastructure for the international competitiveness of the Slovenian RRI space – RI-SI-EPOS” co-financed by the Republic of Slovenia, the Ministry of Education, Science and Sport and the European Union from the European Regional Development Fund. Micro climatic monitoring in show caves is also part of EU Research and Innovation Programme Horizon 2020 project EPOS SP.

Stanka Šebela
