# Supplementary material for: Comparison of historical and current temperatures in show caves (Slovenia)
Source: SN Appl Sci. 2021 Dec 4;4(1):1. doi: 10.1007/s42452-021-04881-1 (PMC8643192; doi:10.1007/s42452-021-04881-1)
Supplement: Supplementary file 7 — Supplementary file7 (DOCX 53 KB) [file 42452_2021_4881_MOESM7_ESM.docx]

AUTHORS' ANSWERS TO REVIEWERS'

**Reviewer #1**: Dear author,

The manuscript "Difference between historical and modern air temperature measurements" brings important data, historical and recent.

I consider the presentation of results adequate, and some suggestions about the style of presentation of your hypothesis and results/discussion/conclusion are in the attached file (with comments).

Authors included your suggestions into the new revised text.

In general I suggest a re-organization of your introduction, dividing the historical data in show caves worldwide from climate change as general.

Introduction is re-organized.

Please, verify the attached file with my suggestions/comments.

Suggestions in attached file are included with additional explications:

**Data and Methodology, line 161**

The site is without human impacts close to it. There is a reception building, which is about 100 m far away and does not work in winter.

**Difference between historical and current air temperature measurements, line 361**

I missed more discussion comparing your data with other results in the literature!

There are no published historical temperature data from other show caves Worldwide. Especially Postojnska Jama has remarkable publication (In Italian) and is in this way unique example.

**Reviewer #2**: This manuscript is an analysis of a potentially interesting question - the degree to which temperature changes due to anthropogenic visitation of show caves can be separated from environmental temperature changes. The review of background literature is thorough, and the use of the available historical records valuable. My concerns fall into two areas:

1. Methodology. There is a statement (page 6, 3rd paragraph) that the modern instrumentation has an accuracy of +/- 0.1 C, but there is no indication of how (or if) this this was calibrated. Did the author simply rely on a manufacturers literature? More importantly, there is no description of the instrumentation used for the historical record, nor the calibration of such instruments. Without calibration, how can the records be compared?

Accuracy of modern T instruments baro divers in caves was regularly compared with additional new callibrated instruments. Baro divers fit well in caves with high humidity and have one of the best reputations for the measurements in extreme environments.

Published studies regarding the old instruments do not give additional informations of the type of thermometers. We belive those were the best instruments in historic period if they were able to record daily measurements, from which mean monthly data have been published. We need to know that T oscillations during one-year period in the cave (Pulpito site in Postojnska Jama) can be < 1.0ºC, so also the historic instrument must have been precise enought for detection such small oscillations.

2) Geomorphology/air flow. There is very little information on the location of the temperature measurement sites with respect to airflow in the caves. This is particularly important with respect to monthly differences - generically, temperate zone caves tend to "exhale" cold air in summer and "inhale" cold air in winter - these reversing airflows need to be taken into account. Of course, specific caves may vary from this general pattern depending on the details of the relevant cave passages - horizontal, descending, ascending, connected (or not) to higher/lower entrances, etc etc.

Different air flow during summer and winter period is typical for show caves presented in this manuscript and has already been published in pevious works but it is not crutial for comparison historical with modern conditions. Actual monitoring sites in Postojnska jama (Pulpito and Sepolcro) are places in the same spot as historical measurements, cave ventilation did not change between historical and recent periods. This manuscript deals with general trends (on a year-long bases) in temperature increases, so the ventilation cannot be different for situation in the relation historical-current period.

In Introduction in the third paragraph there is a basic description about cave ventilation.

**Reviewer #3**: The manuscript presents robust results based on solid methodology, in addition to focusing on poorly studied regions and habitats, revealing the relationship between air temperature and show caves activities. This is in line with the aims and scope of this journal.

Despite this, the conclusions is too long and a continuation of the results and the authors should reduce it and emphasize the main contribution of the results

Conclusions have been reduced with more emphasizing to contribution of the results

**Reviewer #4: See attached file.**

SNAS-D-21-01306 Sebela et al, Review of manuscript

The concept of comparing historic data with modern data in relation to visitor numbers is interesting and obviously apposite. However, the value of this study is quite limited. I find that the authors pay little attention to the representativeness of some of the historic data, and therefore make some conclusions not really supported by the data. Consideration of the quality and veracity of historical data is lacking, and of the representativeness/appropriateness of individual sites. The paper is largely descriptive, essentially just graphing and reporting on temperature changes, with virtually no astute discussion of causes. For example, that the cave is warmest in October, but the outside air warmest in August is explained simply by: “The highest increase in outside temperature in August and in Postojnska Jama in October can be explained by the temperature behaviour in the cave in respect of outside conditions”. This really does not explain anything!

With the new Table 1 (t-test) more significant correlations between historical versus modern T has been discussed.

The best supported conclusion – that temperatures in Postojnska Jama have increased but that this cannot be attributed to visitor numbers – has essentially already been published in Sebela et al

2105 (although with somewhat different data, so this current manuscript adds a little).

With the new corrections we emphasized that air T rise in Postojnska Jama is not only due to increase of outside air T but is accompanied with additional heat brought to the cave by visitors, what is presented with new Fig. 4.

The other cave sites are interesting to include, but the historic data are much less complete and the conclusions not nearly so well supported. For some sites (see details below) the historic data can hardly be considered to be representative, and, at the very least, the extreme limitations of data should be emphasised throughout this manuscript. One of the more interesting results – that one of the caves shows no temperature change over the last almost 100 years in spite of regional external rise – is simply listed, with no discussion or explanation of cause. What is it with this particular cave that the global and regional temperature rise does not affect it? This manuscript fails to explore this.

We added additional explication that no significant air T change in Škocjan Caves is probably due to the special cave morphology of the monitoring site. Even if we have only 15 historical measurements and even if we know that outside air T is increasing (+ visitors are increasing) this part of the cave can preserve stable more-decades long micro-climatic conditions.

The title should be modified to “… in three show caves of Slovenia”… (since only three caves are included and these are all in a single small country - Slovenia).

We updated the title with Slovenia. We do not have more than 3 show caves with historic records in Slovenia.

The abstract is irritatingly non-informative. It is essentially a verbose list of temperature changes (that could be much more easily understood if presented in tabular form), but it includes virtually no analysis of the causes of observed data. I want to know *why* the temp differences in one cave were greatest in October (Postojnska), but in March for another (Predjama) … why one cave (Skocjanske) showed *no* temp change since 1928 … etc.

The only indication of analysis is the last sentence in the abstract (that the increase in temp relates to outside temp) … and the final phrase of the abstract is meaningless “the number of visitors to Postojnska Jama should not be underestimated” – if the temp increase is attributable to outside temp, then the number of visitors is irrelevant … (this same comment applies to the same sentence in line

357).

Abstract is re-written.

It took me some time to realise that the choice of which years of data to include was not just capricious, but rather were the only data available. However, I do question whether only three or four years of data can be considered adequate to represent the modern period or the historic period? (three or four years would be fine for more stable caves; however, it is apparent that some of these caves are very well ventilated and thus open to quite variable conditions). The historic data for Predjama cover only one year and those for Skocjanske comprise only 15 measurements in a single year … is this a fair basis for comparison?

Available historic data are limited and this is emphasized in the text. The best data set is from Postojnska Jama and the obvious T increase at Pulpito site is the most reliable fact. We have modern T data until today but the year 2020 was specific because of the covid-19 pandemic that completly closed all 3 show caves for 6 months. So the best comparison was to the end of 2019 when regular visits took place. The period after 2019 must be subject to a new complex study.

The introduction section is too long – we do not get to the aim of the research until line 149 on page 7. The section from line 77 to 149 (which is a summary of recent global climate changes) seems

to be only tangentially relevant to the topic (do we really need to be told again of Amazonian forest

loss, of Atlantic Meridional overturning, etc.?), and could be omitted. The relevance does not become clear even by the end of the paper, since the very limited discussion in this manuscript never returns to these ideas to tie them in with the results.

Introduction is re-organized.

Accuracy, precision and calibration:

Incorrect use of terms “accuracy” and “precision” e.g., line 189, 192 use the term “accuracy”

with reference to the ±0.25°C error when they mean “precision”. Accuracy is a very significant concept

… a measurement can be highly precise (low error), but not accurate because of faulty calibration, and we have no way of finding out how the old instruments were calibrated … So the old instruments may have been both of lower precision and of lower accuracy. We really need to see some attempt to test calibration or at least a good discussion of this point.

We corrected incorrect term in the text and added more info about calibration.

We need to know that T oscillations during one-year period in the cave (Pulpito site in Postojnska Jama) can be < 1.0ºC, so also the historic instrument must have been precise enought for detection such small oscillations.

Significance of differences:

In general, it is hard to know whether the differences noted are actually significant. We need to know whether the particular years were typical or not. For example, for Postojnska Jama we need to know how the particular years of 1933-1937 fit into the general pattern of regional climatic

variation. What was the pattern of external temps in the decades around the 1933-37 period? How do the particular years 2017-2019 fit into the general decadal pattern? Does the cave temp response to regional temp also include a long-term lag (as would be expected – the rock temperature will change over the longer time-scale). If regional external temps in the period 1986-2015 were “anomalously high” (the implication from this statement being that post-2015 the temperatures dropped back to more normal levels), then the rock temp in the cave may retain that high for some years? so might the high cave temperatures one or two years after the end of the anomalous regional high be remnant?

For the area of Postojna we do not have »true« historical outside meteorological measurements. The closest is the capital Ljubljana, which is 50 km far with different climate conditions, so we cannot use those data for Postojna area too.

Recent studies (Šebela 2021) confirmed that since 2009 some deep cave sites (Postojna and Predjama caves) have increased air T (+ increased outside T + increased visitors). On the same Postojna cave sites as historical measurements we have modern data only from 2016 (Sepolcro) and 2017 (Pulpito). Because Postojna cave is well ventilated (Šebela and Turk 2011) outside air with rised T moves into the cave easily. Only dead-end passages can resist to warm air intrusions for some time.

Postojnska Jama:

Line 242-6 seem to be a direct contradiction of the conclusions of the 2015 paper (Sebela et al

2015) and their 2019 paper (Pipan et al 2019), that external climatic change explains most of the in- cave temp change.

We added new explication.

It is not immediately clear which data went into Table 2 (and lines 248-255), which is the dependent variable, and which the multiple independent variables. Multiple regression implies: (i) one dependent variable (e.g., cave temp difference between the two time periods); (ii) several

independent variables which might explain it (e.g., the only two I can think of here are the outside temp difference, and the visitor number difference, but that is not quite clear in this manuscript); and (iii) the assumption that no/little correlation exists between the independent variables (temperatures at all the other sites in the same, well-ventilated, cave are obviously more-or-less correlated and thus not valid for multiple regression). Anyhow, the R2 value is not as important as the probability value – any R2 *must* be accompanied by the *p* value.

We changed Table numbering and added new Table 1 with t-test for Postojnska Jama, which better explaines relation between old and new data.

The paragraph at line 256-259 suddenly appears with no obvious relationship to the previous paragraph. An introductory sentence might enlighten us as to why we are suddenly told about rising temps in France, and changing visitor numbers in a different cave in a different country ??? Is this supposed to be the Discussion section?

Text is re-written.

Predjama Cave:

Now we move on to the second cave … and we realize that the historical data from this Predjama Cave are limited to a single year – which just happens to be the year 1956, the year noted for the February severe cold wave in Europe. I suggest that this can hardly be considered to be representative, and, at the very least, the extreme limitations of data should be emphasised throughout this manuscript.

This manuscript presents few details of Predjama Cave. However, I see (from Sebela and Turk,

2014) that much of the cave has a high level of ventilation, and therefore is relatively susceptible to external temperature changes. More importantly, from Sebela and Turk 2011, I also learn that this particular site in Predjama cave (the one reported in the current manuscript) has a unique microclimate. The rest of the cave is subject to distinct summer-winter ventilation regimes, and the part visited by tourists is dominated by external temp changes. In contrast, the mean annual air temp at Velika Dvorana does not reflect trends in the external temp and temps at this site in the cave are lower than the mean annual surface temp (by ~3°C). Cold air drains through the cave and pools, such that winter temps at this site stay below 0°C . It warms a little in spring, but stabilizes at 9°C when external temps exceed 15°C and does not respond to higher external temps because the temperature inversion remains intact.

(Should I have had to have read this other publication in order to fully understand the current manuscript?)

Hence my question is: Yes, the impact of human visitation can be excluded, but, because of the complication of the temperature inversion, surely Velika Dvorana is far too complex a site? Looking again at the year of historic data, 1956, wouldn’t the extremely cold winter have had a lingering effect at this site, rendering it colder for longer than normal?

Figure 6 is interpreted by these authors as showing higher temperatures. However, again, without error bars, it is hard to assess significance of slight differences. The variability between the three modern years seems to be just as great as the difference between them and the one year of historic data (from instruments of unknown calibration), which began just at the end of one of the most severe winters of the century (the authors mention that the winter temps next year, 1957, were actually *higher* than modern, which further confirms my suspicion that the year of historic data was a very poor one to choose). Trying to claim a significant difference is milking the data to the extreme, and I suggest that the conclusions are not well supported by the data. I also suggest that picking out the month of March as having been significantly different for one particular year in history compared to a few modern years (e.g., Table 4) is ridiculous – data from one month in 1956 cannot be considered to adequately represent typical spring conditions for the time.

Line 288 … Table 2 does not appear to depict these data. It just shows an R2 value with the date 1942-

2019, but we certainly cannot see the temp as reported by Anelli.

Tables are re-organized and text is updated to better explaine the situation.

The paragraph starting at line 307 is quite confusing. It tells us that the external temps from historic data cannot be compared with modern because the measurement site changed significantly … but then, without any attempt to let us know *if, or how,* the old data were adjusted to take account of the location change, they go ahead to compare historic data with modern! So I have no idea how to view Figure 8. The two curves seem to be largely overlapping (and probably within the error bars, if they had been included) ... but I do not know if these historic data are the original ones taken in the

warmer site in front of the cave rather than the modern site in the forest. This extraordinary section ends with a reiteration that historical temps cannot be compared with modern data (so why on earth should we pay any attention to all that pontificating about particular months when the greatest differences occurred, etc.).

Erros bars are added to graphs and previous Figs. 3, 5 and 7 and 8 are eliminated. Informations that we wanted to present from previous Figs. 3, 5 and 7 and 8 are now presented with 2 new Figures (4 and 6) and with new Table 1 (t-test).

I get the impression that the historic data from this site outside Postojnska Jama are the only

historic measures of outside temperature … and that it is this same outside site against which all the

other caves are compared: therefore the same misgivings apply to the other comparisons of cave

temp with outside temp. My confusion increases when I get to the section on Skocjanske Jame, where the modern outside mean annual temperature appears to have been taken from a government website, rather than from the new forest station near to Postojnska Jama.

Postojnska Jama and Predjama are only 15 km apart and exterior of both have very similar climate conditions so the same outside air T (in front of Postojna Cave for Historic data and in the forest above the Postojna cave for modern data) was used for both caves. We do not have other better data. This is explanied in lines 190-193 (manuscript with track changes)

The observation for Skocjanske Jame that temp has not changed significantly since 1928 is one of the most interesting parts of this manuscript … and yet the explanation is just a single sentence presuming it to be related to the water flow in the cave. Yes, the historic data are rather limited, and thus the comparison with modern temps is not very well founded, so perhaps the authors felt that further explanation was not justified, but they thought the result important enough to include in the abstract. From what I can gather by reading about this cave elsewhere, the site is at the side of a large doline but was quite isolated from the main cave (I cannot tell this from the survey extract in Figure 1). Thus it should be close to the regional mean annual temperature (which for Slovenia has increased about 1.2 °C from 1928 to 2016: https://tradingeconomics.com/slovenia/temperature). So, I immediately wonder why this cave site apparently does not show an equivalent rise.

The truble with Škocjan Caves is that we only have 15 historical measurements in 1 year. But anyway we think that this monitoring location resists to T increase because of cave morphology and this additional explication was added to text.

Fig 1. Add entrances so that we can see how close the measurement sites are to entrances.

Added

Fig 2 … Need to add error bars – we need to see the extent of overlap.

Added

Figures need to have unit of temp shown “Temperature (in °C)”. Placing the axis with vertical text looks awkward. Rotating the axis would allow the full axis label to be shown “Temperature difference (in °C)”

Added and also explained in the Fig captions

Table 1: Do not need to include the same number twice … e.g., visitors to outside shown twice, Sep to outside shown twice, Predjama-Velika to outside shown twice, etc. (i.e., half of the matrix could be eliminated)

Old Table 1, which is now Table 3 is reduced

Line 201 … “compared with” rather than “correlated with”

Corrected

The sentence at line 359-360 is confusing because it is not clear what is meant by the word “as”: … “In Škocjanske Jame, air temperature has not increased significantly in comparison to historical measurements, as is the case in Postojnska Jama and Predjama Cave.” It would be less confusing to

say “In Škocjanske Jame, air temperature has not increased significantly in comparison to historical

measurements, whereas in Postojnska Jama and Predjama Cave it has increased”.

Corrected
