# Supplementary material for: Comparison of historical and current temperatures in show caves (Slovenia)
Source: SN Appl Sci. 2021 Dec 4;4(1):1. doi: 10.1007/s42452-021-04881-1 (PMC8643192; doi:10.1007/s42452-021-04881-1)
Supplement: Supplementary file 6 — Supplementary file6 (DOCX 14 KB) [file 42452_2021_4881_MOESM6_ESM.docx]

Letter to Editor,

Dear Editor,

Thank you for good reviews of our manuscript.

1. I added the co-author dr. Janez Turk because he was activelly included in the re-writting the mauscript (+ Tables + Figures) after the reviews.
2. We eliminated some graphs and added new ones with better information. We re-organized Tables and added new Table 1 for t-test.
3. Answers to reviews are in red in separate file.

Sincerelly

Authors

October 2021
